# Short-term functional outcomes of robotic-assisted TKA are better with functional alignment compared to adjusted mechanical alignment

**DOI:** 10.1051/sicotj/2024002

**Published:** 2024-01-18

**Authors:** Michaud Jeffrey, Philippe Marchand, Pascal Kouyoumdjian, Remy Coulomb

**Affiliations:** 1 Orthopedic and Traumatology Surgery Department, CHU Nîmes, University Montpellier 1, Nîmes Place du Professeur Robert Debré 30029 Nîmes France; 2 Laboratory LMGC, CNRS UMR 5508, University of Montpellier II 860 Rue de St – Priest 34090 Montpellier France

**Keywords:** TKA, Ligament balancing, Adjusted mechanical alignment, MAKO, Functional alignment

## Abstract

*Introduction*: Ligament balancing is essential to the functional outcome of total knee arthroplasty (TKA). The optimal method of alignment remains a controversial issue. The primary objective was to compare the clinical outcomes of TKA between functional and adjusted mechanical alignment techniques. The secondary objectives were to compare bone resection, robotic alignment, and radiological assessment. *Materials and methods*: This was a retrospective case-control series comparing TKA performed with functional alignment (FA) and adjusted mechanical alignment (aMA). Sixty-four FA subjects were matched with 64 aMA controls. These two groups were matched for age, gender, body mass index (BMI), surgeon, and type of frontal deformity. Both surgical procedures were performed using the MAKO^®^ haptic robotic system. Functional scores (Forgotten Joint Score (FJS), Knee Society Score (KSS), and Oxford Knee Score (OKS)) were measured at the final postoperative follow-up. A radiographic evaluation was performed at the same time. *Results*: Mean FJS were respectively 63.4 ± 25.1 [0–100] and 51.2 ± 31.8 [0–100] in FA versus aMA group (*p* = 0.034). Mean OKS were respectively 40.8 ± 6.3 [21–48] and 34.9 ± 11.8 [3–48] in FA versus aMA group (*p* = 0.027). Mean KSS were respectively 184.9 ± 17.0 [126–200] and 175.6 ± 23.1 [102–200] in FA versus aMA group (*p* = 0.02). The main residual symptom was “none” for 73.0% versus 57.8%, “instability” for 6.4% versus 21.9%, “Pain” for 19.1% versus 12.5%, and “effusion” for 1.6% and 7.8% respectively for FA and aMA group (*p* = 0.016). There were 4 complications in the FA group versus 5 in the aMA group (*p* > 0.999). Mean postoperative hip-knee-ankle (HKA) robotic assessment were respectively 177.3° ± 2.0 [172–180] and 178.2° ± 2.0 [173–180] for FA and aMA group (*p* = 0.018). The median difference between HKA robotic and HKA radiological was −3.0° (IQR = 3.0; *p* < 0.001). *Conclusion*: With greater residual deformity and without release, functional alignment showed a statistically significantly better short-term clinical outcome than adjusted mechanical alignment. This difference may not be clinically significant.

## Introduction

Although clinical results of total knee arthroplasty (TKA) are usually excellent, 20% of operated patients report dissatisfaction with residual symptoms [1, 2]. The functional outcomes of TKA are affected by the ability to restore physiological ligament balancing [3]. To achieve this ligament balancing, several alignment methods were available: mechanical, kinematic, functional, or hybrid alignment [4, 5].

Mechanical alignment was the most widely used method for TKA. This concept was introduced in the 1970s, notably by Insall et al. [6]. The aim was to position the components perpendicular to the femoral and tibial adjusted mechanical axes, creating an articular interline perpendicular to the limb’s neutral adjusted mechanical axis. This concept defined a systematic approach in TKA but had its limitations. In large varus cases, it often resulted in a difficult-to-correct lateral femoral imbalance, requiring capsulo-ligamentary release. In the 2000s, Howell concurrently developed kinematic alignment (KA) [7, 8]. This approach was based on new bioadjusted mechanical concepts highlighted, which described the three axes governing patellar and tibial movement relative to the femur: the patellar flexion axis, the tibial flexion-extension axis, and the tibial rotation axis [9]. These fundamentals allowed for implanting a TKA in a patient-specific position with a bone-sparing procedure, preserving the native ligament envelope. However, this can lead to significant deformity of the lower limbs and present a risk of loosening and wear [10].

With the advent of robotics, the use of functional alignment (FA) has become more widespread [11]. Ligament balancing is achieved, using medial and lateral gaps (in millimeters) in full extension and at 90° of flexion [12]. The initial technique [13], based on a systematic model adjusted to bone deformities, has evolved into a specific model adapted to balance the laxity of the soft tissues [14].

The main objective was to compare the clinical outcomes of TKA between two functional and adjusted mechanical alignment techniques. The secondary objectives were to compare bone resection templating, robotic alignment, and radiological assessment.

## Materials and methods

This was a retrospective case-control, single-center, multi-operator (three surgeons with more than 10 years of experience in prosthetic knee surgery) study comparing two groups: functional (FA) or adjusted mechanical alignment (aMA). This study was approved by a local Institutional Review Board (N^o^IRB: 22.03.04).

Patients were selected from the local database, which prospectively collects all robotic-assisted total knee replacements between January 2019 and September 2020. Inclusion criteria were: primary TKA using robotic technology, grade 3 or higher Ahlbäck knee osteoarthritis, and adult patients able to provide informed consent. Exclusion criteria were: inflammatory joint disease or traumatic knee.

**Figure 1:**
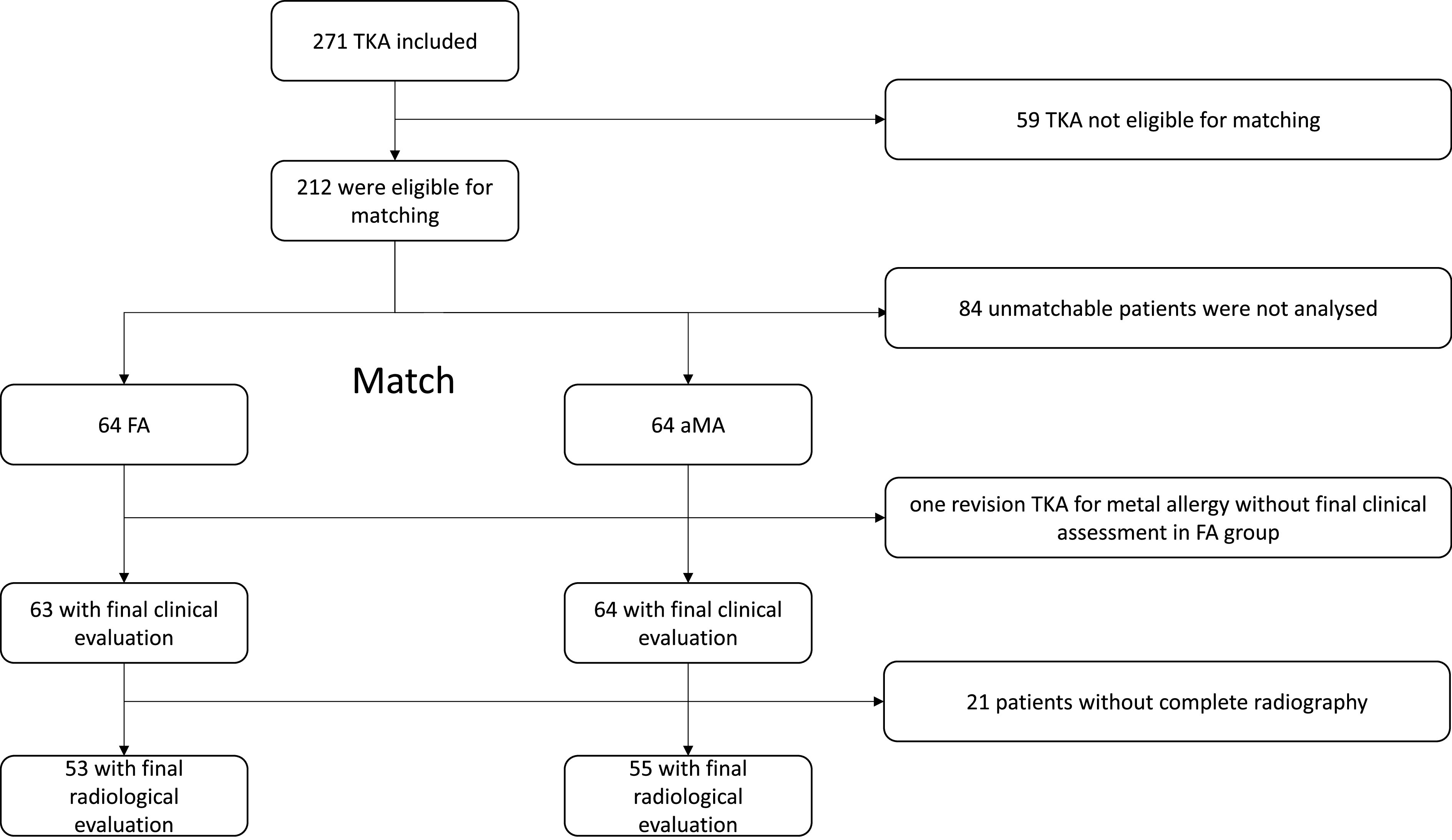
Flow chart. *Abbreviations:* Total Knee Arthroplasty (TKA), Functional Alignment (FA), Adjusted Mechanical Alignment (aMA).

Case-control pairing was performed to create these two balanced cohorts. Each patient was matched with another patient from the opposite group at a 1:1 ratio using propensity score matching, taking into account age, gender, body mass index (BMI), surgeon, and type of frontal deformity. Each matching had a cost, which was set at a maximum of 0.03 per pair. The purpose of this ranking and matching was to have two similar populations in all aspects. Among the 271 TKA performed during the inclusion period, 212 were eligible for matching into two balanced groups. Overall, 128 TKA were matched with 64 in each group. Data from the 84 unmatchable patients were not analyzed. The demographic characteristics of the two cohorts are summarized in Table 1. Both series comprised 64 patients each. The results of the patient selection are shown in Figure 1. There were no significant differences between the groups in terms of age, gender, BMI, American Society of Anesthesiologists (ASA) score, mean radiological preoperative hip-knee-ankle (HKA) angle, and mean follow-up. Demographic data are presented in Table 1.

**Table 1:** Comparative demographic data. *Abbreviations:* body mass index (BMI), American Society of Anesthesiologists (ASA), Hip-Knee-Ankle (HKA).

Variable	Functional	Adjusted mechanical	*p*-Value
	*N* = 64	*N* = 64	
Age at surgery (years)	70.2 ± 7.6 [49.0–87.0]	69.2 ± 8.2 [48.0–86.0]	0.504
Gender			0.721
Male	29 (45.31%)	26 (40.62%)	
Female	35 (54.69%)	38 (59.38%)	
BMI	28.9 ± 5.0 [19.4–42.6]	31.3 ± 7.6 [18.8–50.0]	0.147
ASA score			0.781
1	11 (17.19%)	7 (10.94%)	
2	34 (53.12%)	34 (53.12%)	
3	16 (25.0%)	19 (29.69%)	
4	3 (4.69%)	4 (6.25%)	
Radiological preop HKA angle	174.9 ± 4.4 [165.0–191.0]	173.8 ± 4.9 [165.0–187.0]	0.107
Last follow-up (days)	493.8 ± 181.3 [223.0–923.0]	511.9 ± 134.4 [202.0–846.0]	0.221

All patients received a Triathlon total knee system implant (Stryker^®^, Mahwah, USA) with robotic assistance MAKO^®^ software (Stryker^®^, Mahwah, USA). A preoperative morphological scan with 3D reconstruction was performed, enabling pre-positioning of the implants, adjusting size and alignment to the femoral and tibial adjusted mechanical axes. The MAKO^®^ procedure began with femoral and tibial “bone-morphing” to match the surgical landmarks to the preoperative CT scan. The system assessed HKA angle under different conditions after hip center of rotation, ankle and knee center acquisition. Trans-quadricipital para-patellar approach, either medial or lateral, was chosen based on the deformity. The patella was systematically resurfaced. Enhanced recovery after surgery protocol was used for each patient.

**Figure 2:**
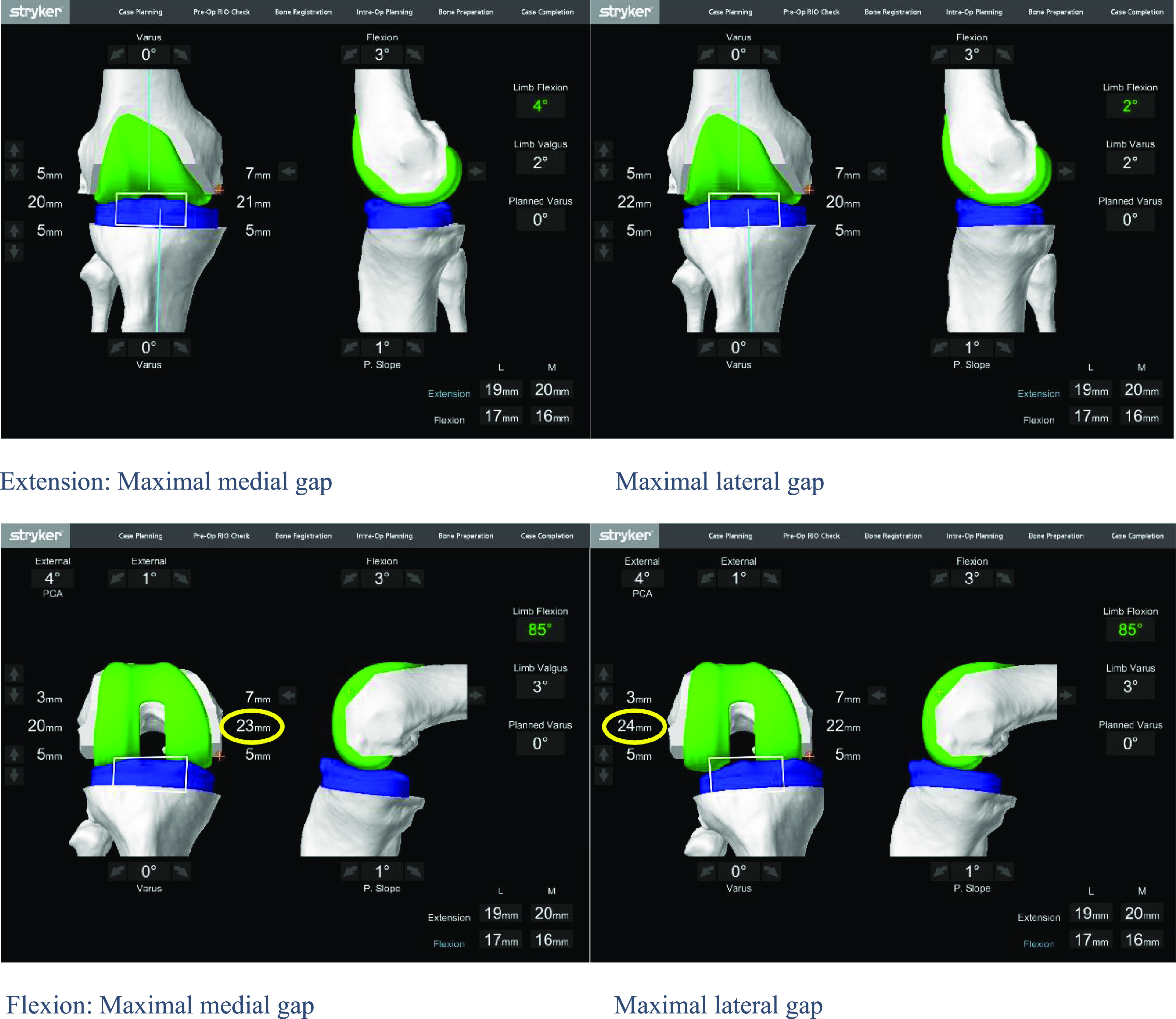
The manoeuvrers allow for estimating the tension of the ligament envelope in extension and flexion.

Functional alignment technique used the ligament balancing. Ligament balancing was assessed by the maximal medial and lateral gaps (in millimeters) in full extension and at 90° of flexion during forced varus and valgus position. These manoeuvrers allowed for estimating the tension of the ligament envelope in extension and flexion (Figure 2). The goal was to modify the bone resection in a way that obtained equal maximal gaps in flexion and extension, close to 18 mm, without considering the overall residual deformity.

Adjusted mechanical alignment was considered a hybrid alignment [5, 15, 16]. It involved unidirectional correction of deformity to achieve an HKA angle as close to 180° as possible. The minimum implant thickness for the Triathlon TKA system was 18 mm ± 1 mm for extreme sizes. This value could vary based on the choice of tibial polyethylene. To balance the medial and lateral gaps in both flexion and extension, bone cuts were adjusted to achieve a residual deformation of less than 3°, and ligament release or pie-crusting could be performed as needed.

Demographic, preoperative clinical and radiological, and preoperative robotic data were collected. Postoperative radio-clinical follow-up was performed at 6 weeks, 4.5 months, and 1 year by each treating surgeon. At the final follow-up, clinical assessment was conducted by an independent observer. Final clinical evaluation was performed for 127 TKA (99%, one revision TKA for metal allergy without final clinical assessment in FA group). Final radiological evaluation was performed for 108 TKA (84.4%, 53 in the FA group and 55 in the aMA group).

Patients filled out a self-questionnaire containing the following scores: Forgotten Joint Score (FJS-12) [17, 18], Oxford Knee Score (OKS) [19], and the subjective portion of the Knee Society Score (KSS) [20]. The FJS-12 was a score ranging from 0 (for a consistently bothersome knee) to 100 (for a completely forgotten knee). The OKS score ranged from 0 (poor result) to 48 (excellent result) and represented the patient’s perspective on knee functionality. The KSS, a composite score (out of 200 points), included an objective knee part filled out by an independent observer (rated from 0 to 100) and a subjective functional part (rated from 0 to 100) filled out by the patient. Knee pain (numeric analogic scale, NAS rated from 0 = no pain to 10 = severe pain), main residual functional symptom (none, pain, pseudo-laxity, effusion), and joint range of motion (ROM) were also recorded at the final follow-up.

Postoperative radiographic measures were done at 1 year on weight-bearing full-length anteroposterior lower limb radiographs and skyline view. Radiographic data: HKA angle, lateral distal femoral angle (lDFA), and medial proximal tibial angle (mPTA) were measured by a single observer using PACS software. An “outliers” measurement corresponded to a patient changing their deformity class, such as initially having a varus (<178°) alignment becoming valgus (>182°) postoperatively or vice versa.

### Statistical analysis

Statistical analysis was performed with EasyMedStat (version 3.29; https://www.easymedstat.com). FJS group comparability was assessed by comparing baseline demographic data and follow-up duration between groups. Normality and hetereoskedasticity of continuous data were assessed with Shapiro-Wilk and Levene’s test respectively. Continuous outcomes were compared with unpaired Student *t*-test, Welch *t*-test, or Mann-Whitney *U*-test according to data distribution. Discrete outcomes were compared with chi-squared or Fisher’s exact test accordingly. The difference between HKA angle robotic and radiologic was assessed with the Wilcoxon signed-rank test. The alpha risk was set to 5% and two-tailed tests were used.

## Results

Mean values of FJS were respectively 63.4 ± 25.1 [0–100] and 51.2 ± 31.8 [0–100] in FA versus aMA group (*p* = 0.034). Mean values of OKS were respectively 40.8 ± 6.3 [21–48] and 34.9 ± 11.8 [3–48] in FA versus aMA group (*p* = 0.027). Mean values of KSS were respectively 184.9 ± 17.0 [126–200] and 175.6 ± 23.1 [102–200] in FA versus aMA group (*p* = 0.02). The main residual symptom was “none” for 74.6% (*n* = 47) versus 57.8% (*n* = 37), “instability” for 6.3% (*n* = 4) versus 21.9% (*n* = 14), “Pain” for 17.5% (*n* = 11) versus 10.9% (*n* = 7) and “effusion” for 1.6% (*n* = 1) and 9.4% (*n* = 6) respectively for FA and aMA group (*p* = 0.016). Clinical outcomes were exposed in Table 2.

**Table 2:** Comparative clinical outcomes.

Variable	Functional	Adjusted mechanical	*p*-Value
	*N* = 64	*N* = 64	
FJS	63.4 ± 25.1	51.2 ± 31.8	0.034
	Range: 0.0; 100.0	Range: 0.0; 100.0	
	*N* = 63	*N* = 64	
OKS	40.8 ± 6.3	34.9 ± 11.8	***0.027***
	Range: 21.0; 48.0	Range: 3.0; 48.0	
	*N* = 63	*N* = 64	
Main functional symptom			***0.017***
Efusion	1 (1.6%)	6 (9.4%)	
Instability	4 (6.3%)	14 (21.9%)	
Pain	11 (17.5%)	7 (10.9%)	
None	47 (74.6%)	37 (57.8%)	
	*N* = 63	*N* = 64	
KSS	184.9 ± 17.0	175.6 ± 23.1	***0.02***
	Range: 126.0; 200.0	Range: 102.0; 200.0	
	*N* = 63	*N* = 64	
KSS knee	94.4 ± 7.76	90.53 ± 14.14	0.514
	Range: 56.0; 100.0	Range: 45.0; 100.0	
	*N* = 63	*N* = 64	
KSS function	90.16 ± 13.88	85.08 ± 15.8	***0.03***
	Range: 45.0; 100.0	Range: 40.0; 100.0	
	*N* = 63	*N* = 64	
NAS knee pain	1.48 ± 1.12	2.02 ± 1.53	0.062
	Range: 0.0; 4.0	Range: 0.0; 6.0	
	*N* = 63	*N* = 62	
Thigh perimeter difference	−0.422 ± 1.26	−0.637 ± 1.58	0.552
	Range: −4.0; 3.0	Range: −5.0; 3.0	
	*N* = 64	*N* = 62	
Time of unipodal static standing	26.9 ± 19.76	21.65 ± 19.74	0.089
	Range: 3.0; 60.0	Range: 3.0; 60.0	
	*N* = 54	*N* = 51	

*Abbreviations:* Forgot Joint Score (FJS), Oxford Knee Score (OKS), Knee Society Score (KSS), numerical analog scale (NAS). *Significative result was written bold and italics.

The difference in NAS, ROM, thigh circumference, quadriceps muscle strength, and one-leg standing test was not significantly different between groups. There were four complications in the FA group (three cases of complex regional pain syndrome and one revision TKA for metal allergy) versus five in the aMA group (two cases of complex regional pain syndrome, one quadricipital tendon tear, and one manipulation under anesthesia for stiffness) (*p* > 0.999).

Femoral, tibial, and patellar implant sizes were not statistically different between both groups. Mean values of polyethylene thickness were respectively 9.5 ± 0.9 [9–13] and 9.94 ± 1.1 [9–13] in FA and Ma group (*p* = 0.016). Intraoperative templated medial tibial, distal medial and lateral femoral, posterior medial femoral thickness resection, femur frontal alignment, femoral flexion or rotation and tibial slope were not statistically different between both groups. Intraoperative templated lateral tibial and posterior lateral femoral resection thickness were lower in the FA group (*p* = 0.041 and *p* = 0.006) while tibia frontal alignment was higher in the FA group (*p* < 0.001). The robotic measures were resumed in Table 3.

**Table 3:** Comparative robotic measures.

Variable	Functional	Mechanical	*p*-Value
	*N* = 64	*N* = 64	
Implant			0.744
CR	60 (93.75%)	58 (90.62%)	
PS	4 (6.25%)	6 (9.38%)	
Femur size	4.14 ± 1.62	3.94 ± 1.45	0.471
	Range: 1.0; 7.0	Range: 1.0; 7.0	
Tibia size	3.97 ± 1.68	3.8 ± 1.47	0.621
	Range: 1.0; 7.0	Range: 1.0; 7.0	
PE thickness	9.5 ± 0.943	9.94 ± 1.13	***0.016***
	Range: 9.0; 13.0	Range: 9.0; 13.0	
Patella size	34.78 ± 3.06	33.84 ± 3.08	0.092
	Range: 29.0; 40.0	Range: 29.0; 42.0	
Tibial medial resection	4.27 ± 1.71	4.22 ± 1.74	0.642
	Range: 1.0; 9.0	Range: 1.5; 11.0	
Tibial lateral resection	5.62 ± 1.84	6.5 ± 1.78	***0.041***
	Range: 0.5; 8.5	Range: 2.0; 11.0	
Femoral medial resection	7.4 ± 1.77	7.36 ± 1.7	0.961
	Range: 2.5; 10.5	Range: 0.8; 10.0	
Femoral lateral resection	5.03 ± 1.84	5.2 ± 1.7	0.595
	Range: 1.0; 10.0	Range: 1.5; 9.0	
Femoral postero-medial resection	8.1 ± 1.33	8.27 ± 1.67	0.335
	Range: 2.5; 10.5	Range: 2.5; 12.0	
Femoral postero-lateral resection	5.22 ± 1.71	5.99 ± 1.33	***0.006***
	Range: 1.5; 9.0	Range: 3.0; 9.0	
Femoral external rotation	3.62 ± 1.85	3.63 ± 1.84	0.981
	Range: 0.0; 6.9	Range: 0.1; 9.8	
Femoral flexion	4.52 ± 1.65	3.91 ± 1.78	0.065
	Range: 1.0; 8.0	Range: 0.0; 7.0	
Tibial slope	1.8 ± 0.87	1.5 ± 0.927	0.231
	Range: 0.0; 3.0	Range: 0.0; 3.0	
Femoral alignment	0.364 ± 0.732	0.329 ± 0.866	0.748
	Range: −1.0; 3.3	Range: −1.0; 4.8	
Tibial alignment	1.31 ± 1.31	0.475 ± 0.853	***<0.001***
	Range: 0.0; 4.0	Range: −1.0; 3.0	
Residual MAKO deformation	2.62 ± 2.19	1.65 ± 2.06	***0.013***
	Range: −4.0; 8.0	Range: −3.0; 7.0	

*Abbreviations:* Cruciate ligament-retaining (CR), posterior-stabilized (PS), Polyethylene (PE). *Significative result was written bold and italics.

**Figure 3:**
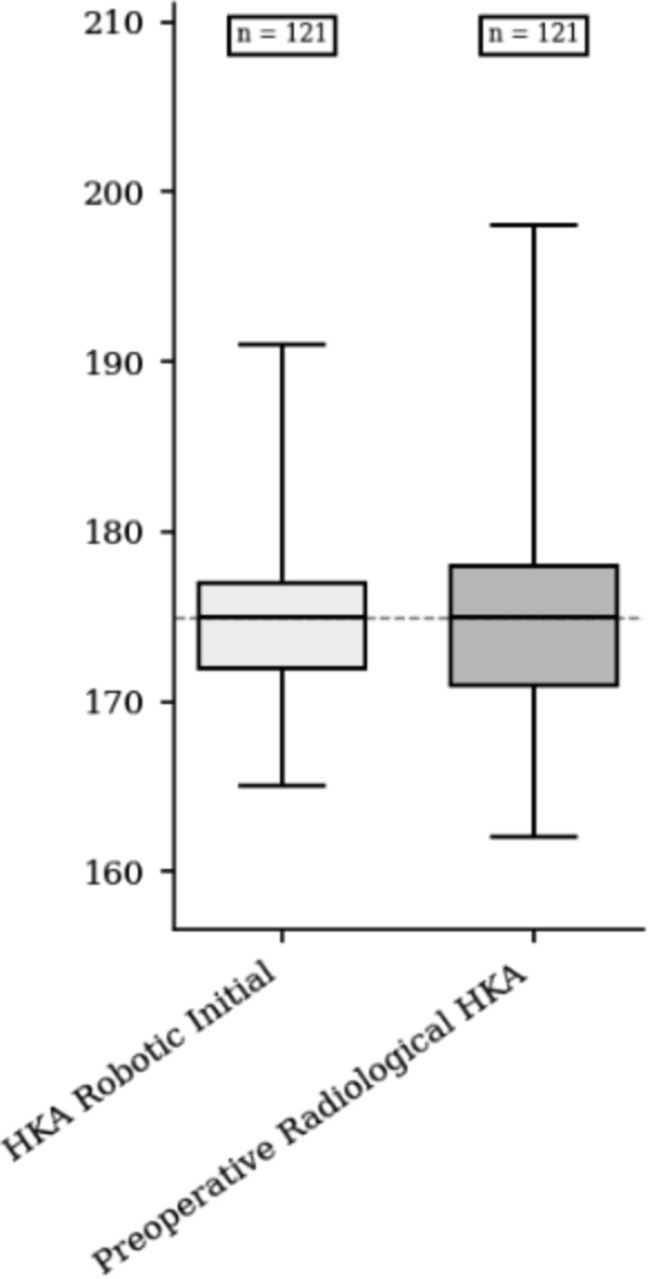
Comparative box plot of preoperative radiological and initial robotic hip-knee-ankle angle.

**Figure 4:**
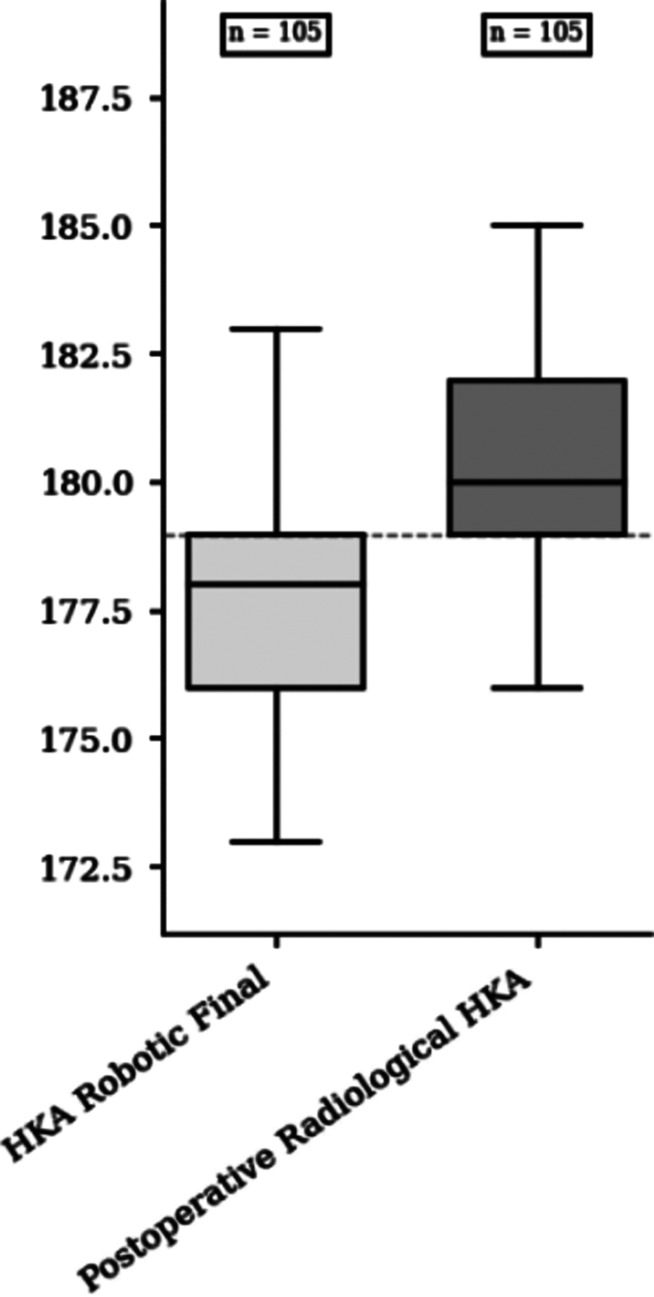
Comparative box plot of postoperative radiological and final robotic hip-knee-ankle angle.

Mean values of postoperative HKA robotic assessment were respectively 177.3° ± 2.0 [172–180] and 178.2° ± 2.0 [173–180] for FA and aMA group (*p* = 0.018). Mean values of postoperative HKA radiological assessment were respectively 180.2° ± 2.2 and 180.6° ± 2.0 for FA and aMA group (*p* = 0.201). Median HKA robotic initial and preoperative radiological HKA were respectively 175.0 (IQR = 5.0) and 175.0 (IQR = 7.0). The median difference was 0.0 (IQR = 2.0; CI95% = [−1.0; 0.0]; *p* = 0.136) (Figure 3). Median HKA robotic and HKA radiological assessment were respectively 178.0° (IQR = 3.0) and 180.0° (IQR = 3.0). The median difference was −3.0° (IQR = 3.0; CI95% = [−3.5; −2.5]; *p* < 0.001) (Figure 4).

Postoperative radiological HKA outliers were significantly different (*p* < 0.0001) between FA (*n* = 3/53, 5.7%) and aMA group (*n* = 14/55, 25.4%).

## Discussion

In a matched population of robotic arm-assisted TKA, this study found better clinical outcomes with a functional versus adjusted mechanical alignment technique. Lateral tibial or postero-lateral femoral bone resections and PE size were lower. In the functional alignment group, residual tibial varus was greater, as was intraoperative deformity, with no difference in overall radiological alignment.

Recently, Parratte et al. [12] found that at 6 months post-op, the knee and function KSS improved (59.3 ± 11.9 and 51.7 ± 20, respectively, versus 49.3 ± 9.7 and 20.8 ± 13; *p* < 0.001) in the functional alignment group compared to the adjusted mechanical alignment group. These differences disappeared at 12 months postoperatively. In the same way, functional alignment with robotic‑arm-assisted TKA demonstrated better patient-reported outcomes than adjusted mechanical alignment with manual total knee arthroplasty [21]. However, it is not clear whether this difference is due to the use of robotics or functional alignment.

It should be noted that the FJS values of this study are significantly lower than the mean FJS-12 values, 89.3 ± 9.2 versus 87.5 ± 12.8 respectively for CS and PS implants, found by Daffara et al. [22] at 24 months post-op. Conversely, the mean FJS scores in this study were much higher than those obtained by Singh et al. [23] at one year post-op (FJS: 42.6 ± 27.8). This underlines the difficulty of comparing studies with a score as subjective as the FJS. Kafelov et al. [24] found the FJS score (76.3 ± 13) similar to our study. Chithartha et al. [25] found a constant evolution of the mean modified FJS from 64.4 at 3 weeks to 89.9 at 2 years, before a progressive decline to 82.7 at 10 years. Kayani et al. [26] noted a significant improvement in the FJS at one-year, two-year, and at five-year follow-ups. The variability in time to FJS follow-up of this study may explain these differences with the literature.

In this study, the difference in mean FJS value between the two groups was around 12 points. This difference was significant. When we use the minimal clinically important difference (MCID) or minimal important change (MIC), as recommended by Clement et al. [27], it would seem that this difference is below clinical significance. Indeed, Clement et al. [27] reported that the MCID for FJS was 16.6 and when the confounding factors were adjusted 13.7. Similarly, the cohort MIC for FJS was 17.7. All these values are higher than the difference found in this study.

In a recent study, Ueyama et al. [28], observed that 23% of a medial-pivot TKA population reported subjective postoperative knee instability. Clinical scores (FJS, KSS), flexion, and satisfaction were lower in this population as well. The rate of subjective instability, more than three times higher in the adjusted mechanical alignment group, could explain the poorer results. In contrast to the statements made by Marchand et al. [29], the use of preoperative CT scans and the precision of robot-assisted TKA planning do not always result in well-balanced knees. This underlines the relevance of a personalized alignment [4].

Regarding alignment, residual deformity was more significant with less outliers deformity in the functional alignment group. Abdel et al. [30] showed that in the long-term, a neutral limb alignment did not guarantee a functional result and better implant survival. Similarly, Kaneko et al. [31] found that robotic TKA resulted in various HKA, femoral mechanical axis (FMA), and tibial mechanical axis (TMA) phenotypes in the coronal plane, none of which affected PROMs. With regard to the difference between intraoperative MAKO and postoperative radiological HKA measurements, it is interesting to note that Glowalla et al. [32] recently made the same observation. The authors found that intraoperative HKA assessment with definitive implants showed a mean residual varus deformity of 3.2° ± 1.9°, whereas a significantly lower residual varus deformity of 1.4° ± 1.9° was identified in the postoperative FLR (*p* < 0.001). This result is very similar to the present study. The authors suggest considering this difference to avoid overcorrecting TKAs for knees with low deformity. This probably explains the outlier rate in the adjusted mechanical alignment group.

This study has several limitations. First, the data collection is prospective, but the analysis is retrospective. Matching does not allow a continuous series like a randomized therapeutic trial. In addition, as the evolution of practices has gradually moved away from an adjusted mechanical philosophy towards functional alignment, the two series are not contemporary. Secondly, despite a uniform distribution of deformity classes (varus/neutral/valgus) in each group, a detailed analysis of each morphotype was not possible due to limited statistical power. The low presence of valgus (5 pairs) and neutral (12 pairs) in each group is a limiting factor and does not allow subgroup analysis. Third, the usual clinical follow-up was a consultation at 6 weeks, 4.5 months, 1 year, and then every 5 years. Unfortunately, some patients did not return for consultation 1 year postoperatively and others returned between 1 and 3 years to take care of the second side. This explains the difference in follow-up of almost 2 years, although not significant between the groups. Finally, the radiological description of patients is based on measurements carried out by a single observer. However, these measurements are performed on weight-bearing radiographs where flexion, limb rotation, and sagittal balance are not controlled. Ilahi et al. [33] demonstrated intraobserver variability of less than 4° on standard radiographs. Lonner et al. [34] demonstrated the significant influence of limb positioning, with measurement variability of up to 8° during radiographic evaluation. Therefore, all these factors may have biased the pre- and postoperative radiographic measurements. In addition, no analysis of the obliquity of the joint line was carried out.

A key strength of our study is that many data points are based on morphological data measured by the robotic system, therefore robust and identical between groups. Few studies compare two surgical techniques using robotics and surgical measures. Maintaining comparability between groups through matching controlled for confounding factors is a strength. The comparison of two different techniques with the same surgical instrument carried out at a distance from our learning curve reinforced our results.

## Conclusion

With greater residual deformity and without release, functional alignment showed a statistically significantly better short-term clinical outcome than adjusted mechanical alignment. This difference may not be clinically significant. A systematic analysis with longer follow-up is necessary to confirm these results.

## Conflicts of interest

Michaud Jeffrey declares no financial interests.

## Financial interests

Philippe Marchand and Remy Coulomb have received consultant honoraria from Stryker. Pascal Kouyoumdjian has received consultant honoraria and speaker honorarium from Stryker and Lepine.

## Funding

This research did not receive any specific grant from funding agencies in the public, commercial, or not-for-profit sectors. Prosthetic implants and robot MAKO^®^ were provided by Stryker.

## Ethical approval

This study was approved by a local Institutional Review Board (N^o^IRB: 22.03.04).

## Informed consent

Informed consent was obtained from adult patients.

## Author contributions

Michaud Jeffrey: Investigation, Writing, Editing.

Philipe Marchand: Methodology, Investigation, Reviewing.

Pascal Kouyoumdjian: Supervision, Reviewing.

Remy Coulomb: Methodology, Investigation, Writing, Reviewing.

## References

[1] Bourne RB, Chesworth BM, Davis AM, et al. (2010) Patient satisfaction after total knee arthroplasty: who is satisfied and who is not? Clin Orthop Relat Res 468, 57–63. 19844772 10.1007/s11999-009-1119-9 PMC2795819

[2] Canovas F, Dagneaux L (2018) Quality of life after total knee arthroplasty. Orthop Traumatol Surg Res 104, S41–S46. 29183821 10.1016/j.otsr.2017.04.017

[3] Jaffe WL, Dundon JM, Camus T (2018) Alignment and balance methods in total knee arthroplasty. J Am Acad Orthop Surg 26, 709–716. 30134305 10.5435/JAAOS-D-16-00428

[4] Lustig S, Sappey-Marinier E, Fary C, et al. (2021) Personalized alignment in total knee arthroplasty: current concepts. SICOT J 7, 19. 33812467 10.1051/sicotj/2021021 PMC8019550

[5] Rivière C, Iranpour F, Auvinet E, et al. (2017) Alignment options for total knee arthroplasty: A systematic review. Orthop Traumatol Surg Res 103, 1047–1056. 28864235 10.1016/j.otsr.2017.07.010

[6] Insall JN, Binazzi R, Soudry M, Mestriner LA (1985) Total knee arthroplasty. Clin Orthop Relat Res 192, 13–22. 3967412

[7] Lee YS, Howell SM, Won Y-Y, et al. (2017) Kinematic alignment is a possible alternative to mechanical alignment in total knee arthroplasty. Knee Surg Sports Traumatol Arthrosc 25, 3467–3479. 28439636 10.1007/s00167-017-4558-y

[8] Rivière C, Harman C, Boughton O, Cobb J (2020). The kinematic alignment technique for total knee arthroplasty. In: Personalized hip and knee joint replacement. Rivière C, Vendittoli PA, Editors. Cham (CH), Springer. 33347122

[9] Eckhoff DG, Bach JM, Spitzer VM, et al. (2005) Three-dimensional mechanics, kinematics, and morphology of the knee viewed in virtual reality. J Bone Joint Surg Am 87 (Suppl 2): 71–80. 16326726 10.2106/JBJS.E.00440

[10] Hiranaka T, Suda Y, Saitoh A, et al. (2022) Current concept of kinematic alignment total knee arthroplasty and its derivatives. Bone Jt Open 3, 390–397. 35532356 10.1302/2633-1462.35.BJO-2022-0021.R2 PMC9134837

[11] Shatrov J, Foissey C, Kafelov M, et al. (2023) Functional alignment philosophy in total knee arthroplasty-rationale and technique for the valgus morphotype using an image based robotic platform and individualized planning. J Pers Med 13, 212. 36836446 10.3390/jpm13020212 PMC9961945

[12] Parratte S, Van Overschelde P, Bandi M, et al. (2023) An anatomo-functional implant positioning technique with robotic assistance for primary TKA allows the restoration of the native knee alignment and a natural functional ligament pattern, with a faster recovery at 6 months compared to an adjusted mechanical technique. Knee Surg Sports Traumatol Arthrosc 31, 1334–1346. 35552475 10.1007/s00167-022-06995-4

[13] Oussedik S, Abdel MP, Victor J, et al. (2020) Alignment in total knee arthroplasty. Bone Joint J 102-B, 276–279. 32114811 10.1302/0301-620X.102B3.BJJ-2019-1729

[14] Shatrov J, Battelier C, Sappey-Marinier E, et al. (2022) Functional alignment philosophy in total knee arthroplasty – rationale and technique for the varus morphotype using a CT based robotic platform and individualized planning. SICOT J 8, 11. 35363136 10.1051/sicotj/2022010 PMC8973302

[15] De Muylder J, Victor J, Cornu O, et al. (2015) Total knee arthroplasty in patients with substantial deformities using primary knee components. Knee Surg Sports Traumatol Arthrosc 23, 3653–3659. 25246172 10.1007/s00167-014-3269-x

[16] Vanlommel L, Vanlommel J, Claes S, Bellemans J (2013) Slight undercorrection following total knee arthroplasty results in superior clinical outcomes in varus knees. Knee Surg Sports Traumatol Arthrosc 21, 2325–2330. 23552665 10.1007/s00167-013-2481-4

[17] Giesinger JM, Behrend H, Hamilton DF, et al. (2019) Normative values for the forgotten joint score-12 for the US general population. J Arthroplasty 34, 650–655. 30612834 10.1016/j.arth.2018.12.011

[18] Klouche S, Giesinger JM, Sariali E (2018) Traduction et validation transculturelle du score de l’articulation oubliée (Forgotten Joint Score) dans les prothèses totales de hanche. Rev Chir Orthop Traumatol 104, 466–470.

[19] Gummaraju A, Maillot C, Baryeh K, et al. (2021) Le score Oxford Knee et l’EQ-5d prédisent mal la satisfaction du patient après une arthroplastie totale du genou suivant un alignement mécanique: une étude transversale. Rev Chir Orthop Traumatol 107, 308.

[20] Debette C, Parratte S, Maucort-Boulch D, et al. (2014) French adaptation of the new Knee Society Scoring System for total knee arthroplasty. Orthop Traumatol Surg Res 100, 531–534. 25082773 10.1016/j.otsr.2014.03.025

[21] Choi BS, Kim SE, Yang M, et al. (2023) Functional alignment with robotic-arm assisted total knee arthroplasty demonstrated better patient-reported outcomes than mechanical alignment with manual total knee arthroplasty. Knee Surg Sports Traumatol Arthrosc 31, 1072–1080. 36378291 10.1007/s00167-022-07227-5

[22] Daffara V, Zambianchi F, Bazzan G, et al. (2023) No difference in clinical outcomes between functionally aligned cruciate-retaining and posterior-stabilized robotic-assisted total knee arthroplasty. Int Orthop 47, 711–717. 36648533 10.1007/s00264-023-05693-1

[23] Singh V, Fiedler B, Huang S, et al. (2022) Patient acceptable symptom state for the forgotten joint score in primary total knee arthroplasty. J Arthroplasty 37, 1557–1561. 35346809 10.1016/j.arth.2022.03.069

[24] Kafelov M, Batailler C, Shatrov J, et al. (2023) Functional positioning principles for image-based robotic-assisted TKA achieved a higher Forgotten Joint Score at 1 year compared to conventional TKA with restricted kinematic alignment. Knee Surg Sports Traumatol Arthrosc 31, 5591–5602. 37851026 10.1007/s00167-023-07609-3

[25] Chithartha K, Nair AS, Thilak J (2021) A long-term cross-sectional study with modified forgotten joint score to assess the perception of artificial joint after total knee arthroplasty. SICOT J 7, 14. 33704059 10.1051/sicotj/2021013 PMC7949890

[26] Kayani B, Fontalis A, Haddad IC, et al. (2023) Robotic-arm assisted total knee arthroplasty is associated with comparable functional outcomes but improved forgotten joint scores compared with conventional manual total knee arthroplasty at five-year follow-up. Knee Surg Sports Traumatol Arthrosc 31, 5453–5462. 37804346 10.1007/s00167-023-07578-7

[27] Clement ND, Scott CEH, Hamilton DF, et al. (2021) Meaningful values in the Forgotten Joint Score after total knee arthroplasty: minimal clinical important difference, minimal important and detectable changes, and patient-acceptable symptom state. Bone Joint J 103-B, 846–854. 33934639 10.1302/0301-620X.103B5.BJJ-2020-0396.R1

[28] Ueyama H, Kanemoto N, Minoda Y, et al. (2022) Association of a wider medial gap (medial laxity) in flexion with self-reported knee instability after medial-pivot total knee arthroplasty. J Bone Joint Surg Am 104, 910–918. 35320136 10.2106/JBJS.21.01034

[29] Marchand RC, Sodhi N, Bhowmik-Stoker M, et al. (2019) Does the robotic arm and preoperative ct planning help with 3d intraoperative total knee arthroplasty planning? J Knee Surg 32, 742–749. 30112739 10.1055/s-0038-1668122

[30] Abdel MP, Ollivier M, Parratte S, et al. (2018) Effect of postoperative mechanical axis alignment on survival and functional outcomes of modern total knee arthroplasties with cement: a concise follow-up at 20 years. J Bone Joint Surg Am 100, 472–478. 29557863 10.2106/JBJS.16.01587

[31] Kaneko T, Yamamoto A, Takada K, Yoshizawa S (2023) Coronal alignment classes after robotic-assisted total knee arthroplasty are not associated with variation in patient-reported outcome measurements: A single-center cohort study. Knee 41, 274–282. 36774917 10.1016/j.knee.2023.01.015

[32] Glowalla C, Langer S, Lenze U, et al. (2023) Postoperative full leg radiographs exhibit less residual coronal varus deformity compared to intraoperative measurements in robotic arm-assisted total knee arthroplasty with the MAKO^TM^ system. Knee Surg Sports Traumatol Arthrosc 31, 3912–3918. 36964782 10.1007/s00167-023-07386-z PMC10435414

[33] Ilahi OA, Kadakia NR, Huo MH (2001) Inter- and intraobserver variability of radiographic measurements of knee alignment. Am J Knee Surg 14, 238–242. 11703037

[34] Lonner JH, Laird MT, Stuchin SA (1996) Effect of rotation and knee flexion on radiographic alignment in total knee arthroplasties. Clin Orthop Relat Res 331, 102–106. 10.1097/00003086-199610000-00014 8895625

